# Adverse Childhood Experiences and the Consequences on Neurobiological, Psychosocial, and Somatic Conditions Across the Lifespan

**DOI:** 10.3389/fpsyt.2018.00420

**Published:** 2018-09-04

**Authors:** Julia I. Herzog, Christian Schmahl

**Affiliations:** ^1^Department of Psychosomatic Medicine and Psychotherapy, Central Institute of Mental Health Mannheim, Medical Faculty Mannheim, Heidelberg University, Mannheim, Germany; ^2^Department of Psychiatry, Schulich School of Medicine and Dentistry, Western University, London, ON, Canada

**Keywords:** adverse childhood experiences, childhood maltreatment, neuroimaging, psychopathology, somatic disorders, type and timing

## Abstract

**Introduction:** Adverse childhood experiences (ACE) such as sexual and physical abuse or neglect are frequent in childhood and constitute a massive stressor with long-lasting adverse effects on the brain, mental and physical health.The aim of this qualitative review is to present a concise overview of the present literature on the impact of ACE on neurobiology, mental and somatic health in later adulthood.

**Methods:** The authors reviewed the existing literature on the impact of ACE on neurobiology, mental and somatic health in later adulthood and summarized the results for a concise qualitative overview.

**Results:** In adulthood, the history of ACE can result in complex clinical profiles with several co-occurring mental and somatic disorders such as posttraumatic stress disorder, depression, borderline personality disorder, obesity and diabetes. Although a general stress effect in the development of the disorders and neural alterations can be assumed, the role of type and timing of ACE is of particular interest in terms of prevention and treatment of ACE-related mental and somatic conditions. It has been suggested that during certain vulnerable developmental phases the risk for subsequent ACE-related disorders is increased. Moreover, emerging evidence points to sensitive periods and specificity of ACE-subtypes in the development of neurobiological alterations, e.g., volumetric and functional changes in the amygdala and hippocampus.

**Conclusion:** Longitudinal studies are needed to investigate complex ACE-related characteristics and mechanisms relevant for mental and somatic disorders by integrating *state of the art* knowledge and methods. By identifying and validating psychosocial and somatic risk factors and diagnostic markers one might improve the development of innovative somatic and psychological treatment options for individuals suffering from ACE-related disorders.

## Introduction

Converging evidence from epidemiological and neurobiological studies suggest adverse childhood experiences (ACE) such as sexual and physical abuse and related adverse experiences to be closely related to enduring brain dysfunctions that, in turn, affect physical and mental health throughout the lifespan (1–3). This is particularly relevant, since community surveys from Europe and worldwide show high prevalence rates of physical (22.9%), emotional (29.1%) and sexual (9.6%) abuse, as well as physical (16.3%) and emotional neglect (18.4%) (4). Unfortunately, these numbers probably under-represents the real dimension of the problem, as they do not account for the high number of unreported cases. Individuals with ACE seem to be at higher risk for the development of mental and somatic disorders throughout the lifespan (2, 5–8). Psychological and psychosocial mechanisms known to contribute to mental disorders are affected after ACE, comprising disturbances in cognitive and affective processing such as for example heightened attention toward threatening stimuli (9). These alterations are mirrored in functional alterations in key stress–and emotion associated brain regions (anterior cingulate cortex [ACC], amygdala, hippocampus) (9–17) [for review see: (18–20)]. Importantly, these are not restricted to functional, but also mirrored in morphometric changes: Volumetric alterations in amygdala, hippocampal, as well as in the ACC have been reported in several investigations (9, 13, 21–27). Several etiological models describe neural alterations and symptom severity usually with a linear function (dose-dependent effect) of cumulative ACE load (multiplicity/number of event types) (3, 28–32) or overall severity of exposure (33)). Next to the dose-dependent effect, studies suggest a different impact of type and timing of ACE in terms of neurobiological alterations, mental and somatic consequences (26, 30, 34, 35). The aim of this qualitative review is to present a concise overview of the present literature on the impact of ACE on neurobiology, mental, and somatic health in later adulthood. Therefore, we searched the databases PubMed, Web of science, PsycINFO for literature on the impact of ACE on neurobiology, mental, and somatic health in later adulthood. We included manuscripts based on original research as well as reviews and meta-analyses and summarized the results for a concise qualitative overview.

### ACE-structural and functional brain alterations and the role of type and timing of exposure

At the present time, there is clear evidence that ACE and ACE-related disorders are associated with enduring effects on the structure and function of neural stress-regulatory circuits such as for example the hippocampus, the amygdala or the ACC (35, 36) and promote alterations in stress sensitivity and emotion regulation in later life. The respective brain regions could be especially vulnerable to the impact of ACE due to a high density of glucorticoid receptors and high vulnerability to the effects of glucorticoids via damage, dendritic atrophy and neurogenesis suppression (37, 38).

#### Structural neuroimaging studies

On balance, many studies point at reduced volume of the hippocampus in individuals after ACE compared to non-maltreated individuals (9, 27, 34, 39–45). However, a recent meta-analysis showed that this difference becomes less evident when controlling for gender (37). Moreover, several studies demonstrated greater hippocampus reduction in males than females (46–48), suggesting that the increased resilience in women may be associated with a protective effect of estrogen. For those readers interested in the latter, we would recommend the review by Helpman et al. (49) and a recent study by Teicher et al. (50). The amygdala has, next to the hippocampus, a high density of glucocorticoid receptors on stress-susceptible pyramidal cells (36). Interestingly, opposite to the effects of stress on the hippocampus, stress stimulates dendritic arborization on pyramid cells in the amygdala, leading to increasing volume (36, 51). However, studies of ACE on amygdala volume are controversial. Several studies have reported increased amygdala volume in institutionally reared children (13, 52), children with chronically depressed mothers (53) and adult subjects with disturbed attachment bonds as infants (26). Contrary, reduced amygdala volumes were found in adults after ACE with diagnoses of BPD (41, 43, 54), Dissociative Identity Disorder (55) and substance abuse (56). Hence, these results are broadly consistent with the hypothesis that amygdala hypertrophy may be more related to early exposure to emotional and/or physical neglect, whereas decreased amygdala volume rather is reported in adults or older adolescents with a greater degree of psychopathology (e.g., BPD) and with exposure to multiple and very severe abuse (26, 36). Numerous studies have reported attenuated development of the ACC after ACE, for example reduced ACC volume (21, 57) and diminished thickness (58).

Several studies have demonstrated that exposure to specific types of ACE selectively affect sensory systems, which were involved in perceiving the trauma that was experienced (35, 36). For example, the exposure to parental verbal abuse seems to significantly target the arcuate fasciculus, a region which interconnects Broca and Wernicke's area (59). Moreover, witnessing domestic violence seems to affect the inferior longitudinal fasciculus (60), that interconnects visual and limbic systems (35). In line with this, Tomoda et al. (61) reported reduced visual cortex and right lingual gyrus gray matter volume in young adults who were exposed to witnessing domestic violence in childhood. The respective regions were maximally sensitive to exposure between the ages of 11–13 years (61). Heim et al. (58) investigated cortical thickness in healthy women who were exposed to childhood maltreatment. The authors found, that exposure to childhood sexual abuse was associated with thinning of the somatosensory genital field. Contrary, women who were exposed to emotional abuse showed thinning in brain regions associated with self-awareness and self-evaluation. Andersen et al. (34) investigated sensitive time periods of trauma exposure in women with a history of childhood sexual abuse. In this case, the hippocampal volume was found to be maximally susceptible to sexual abuse when individuals were exposed between the ages of 3 and 5 years (34). A study by Pechtel et al. (26) compared healthy controls with exposure to emotional abuse and neglect with healthy controls without a history of ACE and found evidence for increased bilateral amygdala volume. Using the Maltreatment and Abuse Chronology of Exposure Scale [MACE; (62)] for sensitive period analyses, the authors showed that the right amygdala appeared to be most sensitive to maltreatment at 10–11 years of age. In a longitudinal study of subjects with ACE, Whittle et al. (63) reported data of a linear effect of ACE on left amygdala development, such that higher levels of ACE were associated with a suppressive effect on amygdala development over time. These studies are in line with the hypothesis of an initial increase in amygdala volume after ACE, followed by a decrease in volume due to persistent and severe maltreatment in later life (36).

#### Functional neuroimaging studies

In contrast to the high amount of studies investigating structural brain alterations, only a few have examined functional differences related to ACE. Fortunately, over the last decade, an increasing number of studies have been published using functional neuroimaging (fMRI) techniques to examine possible associations between ACE and alterations in neurocognitive systems.

Evidence from animal and human research proposes that the amygdala is critically involved in the detection and processing of salient stimuli, especially in those related to danger (64). Neuroimaging research mainly documents amygdala hyperactivity in response to emotional stimuli in individuals with a history of childhood maltreatment (18, 65–69) [for meta-analysis see: (70)]. As this region is highly involved in emotional processes, such as salient detection (esp. stimuli associated with danger) and emotional responses, one can suppose that hyperactivity in this region may be associated with greater risk for the development of dysfunctional behaviors (67). In line with this, hyperactivity in the amygdala is linked to several disorders, including posttraumatic stress disorder (PTSD) (71), anxiety and mood disorders (72) or BPD (54). Moreover, preliminary results suggest that hyperactivation of the amygdala may predict the likelihood of future symptomatology (67, 73, 74). In contrast, several studies reported no differences or even reduced amygdala activity in individuals with ACE compared to control groups when exposed to negative/trauma-related stimuli (75–77). This pattern of hypo-activity to threat-related cues has repeatedly been found in patients with PTSD and severe dissociative symptomatology, pointing toward a specific dissociative subtype in PTSD [for further details please see for example: (78)]. Beyond the amygdala, the anterior insula has found to be hyperactive in individuals with ACE when exposed to emotional stimuli compared to neutral stimuli (66, 70). The anterior insula is associated with the detection of salient stimuli and is assumed to be important for effective modulation of attention in the presence of emotional stimuli (79). Like the amygdala, hyperactivity in the anterior insula to trauma-related stimuli has been found in several mental disorders, such as in social anxiety, specific phobia (72) and PTSD (75). Additionally, a recent meta-analysis of 20 studies of emotion processing in maltreatment individuals, revealed that ACE was (next to hyperactivation in the amygdala and insula), associated with hyperactivation in the superior temporal gyrus, and the parahippocampal gyrus (70). The authors suggest that increased activation in the superior temporal gyrus may aid in early detection of threatening stimuli, which may be an adaptive ability in the context of childhood maltreatment. With regard to the hippocampal formation, evidence suggests that alterations in this system are associated with the development of PTSD. However it is unclear if altered hippocampal formation activation may be a risk factor that interacts with ACE to produce PTSD or a consequence of ACE that predispose an individual to later PTSD (70). The literature on functional alterations of the hippocampal formation in PTSD is mixed: hippocampal activation has been found to be both increased and decreased and parahippocampal activation tends to be increased (80). To our knowledge, studies so far did not systematically contrast different types or timing periods of ACE directly in terms of functional brain differences, which would be needed to further characterize the impact of ACE on brain development.

### ACE and psychosocial consequences

ACE are supposed to affect psychological and psychosocial mechanisms known to contribute to mental disorders, comprising disturbances in cognitive and affective processing such as a heightened attention toward threatening stimuli (9), heightened experience of loneliness (81), as well as social cognitive functioning (19) and social interactions (82) including aggressive behaviors (83). The risk for developing a mental disorder after ACE (in a dose-dependent manner) is highest for depression (3, 84, 85), PTSD (86, 87), borderline personality disorder (BPD) (88), and substance abuse (7, 89). There is growing recognition that individuals with a history of ACE who present with a mental disorder, vary in several respects from individuals within the same diagnostic category without ACE and may therefore represent a specific ecophenotype (35, 67). For example, mental disorders in individuals with ACE are supposed to develop earlier accompanied by more severe symptomatology (90), increased risk of comorbidity (91) and are less likely to respond to standard treatments (92).

A few studies provide evidence that specific types of maltreatment are associated with greater risk for developing psychopathology than other types. In a meta-analysis, Norman et al. (93) reported higher odds ratio for depression when exposed to emotional abuse compared to physical abuse and higher odds ratio for drug abuse when exposed to physical abuse compared to emotional abuse. There is controversial evidence regarding the association between timing of maltreatment and psychopathology. Schoedl et al. (94) examined the relationship between the age of trauma exposure (sexual abuse) and the development of PTSD and depression. The main findings suggest an association between the age of trauma exposure and the likelihood for developing severe depressive or PTSD symptoms in adulthood: Those with earlier exposure (sexual abuse before the age of 12) were at greater risk of having prominent depressive symptoms, while those reporting sexual abuse after the age of 12 were at greater risk of having severe PTSD symptoms. Others have found the opposite effect of maltreatment time and psychopathology (95). Using sensitive time period analyses, Schalinski et al. (30) recently demonstrated that dissociative symptoms (i.e., depersonalization and derealization) were associated with peak vulnerability at 13–14 years of age with emotional neglect being the leading influence followed by other forms of emotional abuse [for further findings regarding clinical outcomes see: (30, 96)]. Moreover, evidence suggest that trauma exposure between 3 and 5 years of age is associated with higher risk for developing PTSD (97), depression and suicidality (98) compared to exposure at 0–2 or 6–8 years. These studies provide evidence that sensitive time periods of risk for psychopathology could be easily missed when comparing broader time frames or overall severity of exposure (30, 96). Finally we have to take into account the question of resilience: Why do two individuals who have experienced very similar patterns of ACE often show very different outcomes? This may be partly due to social environmental or psychological factors but also very likely in part at least due to genetic differences and epigenetic mechanisms. Emerging data suggest that epigenetic mechanisms help to explain the association between ACE and later health problems (99). Researchers have focused on the way in which genetic variants and adverse social environments can interact (gene by environment interaction, GxE) and have shown that a child‘s genotype may partly determine their level of risk and resilience for later psychopathology following ACE including depression (100), bipolar disorder (101) and PTSD (102). Nevertheless, it should be kept in mind that positive environmental influences (e.g., familiar support) can also promote resilience, even in those children carrying “risk” polymorphisms exposed to ACE (103). For further details, we refer to Turecki et al. (99) or Zannas and West (104).

### ACE and somatic consequences

Emerging evidence suggests that ACE is associated with the development of a wide range of somatic disorders, such as obesity, diabetes, inflammatory bowel diseases (IBD) such as ulcerative colitis (UC) and Crohn Disease (CD) (2, 6, 105–107) as well as abnormal pain perception with and without corresponding somatic pathology (e.g., chronic pain vs. pain during child birth) (108, 109).

Recent studies have suggested a dysregulation of the innate immune system as a possible biological mediator between ACE and adulthood disease. These studies have reported an association between ACE and increased levels of pro-inflammatory markers, most notably of the acute phase protein C-reactive protein (CRP), the cytokines interleukin-6 (IL-6) and tumor necrosis factor-α (TNF-α) (110). IBD are driven by the interaction of the intestinal microflora and environmental factors in a genetically susceptible host. Since childhood is a critical phase for the development of neurobiological systems relevant for IBD, e.g., the mucosal immune system, the intestinal microbiota, and immune tolerance in the gut, ACE may contribute to dysfunctions and this effect may depend on the type and timing of ACE. In light of several non-significant findings as well as a significant amount of heterogeneity in methods, Baumeister et al. (111) examined in a recent meta-analysis, whether early-life adversity contributes to potentially pathogenic pro-inflammatory phenotypes in adult individuals. They found a significant association between childhood trauma and elevated baseline peripheral levels of CRP, IL-6 and TNF-α. They conclude that these results provide strong evidence for ACE significantly impacting on the inflammatory immune system, with trajectories reaching into adulthood, thus offering a potential molecular pathway by which early trauma confers vulnerability to developing mental and somatic disorders later in life. Moreover, subgroup analyses for specific types of trauma (sexual, physical or emotional abuse) revealed that these differentially impact specific inflammatory markers. These results indicate that ACE contribute to a pro-inflammatory state in adulthood, with specific inflammatory profiles depending on the specific type of trauma. Furthermore, ACE are known as predictors for chronicity of low back pain and are associated with enhanced pressure pain sensitivity (112). More precisely, early life social stressors such as emotional abuse are associated with enhanced temporal summation of pain and enhanced touch sensitivity (109). Recent animal experiments indicate that stress itself is able to sensitize nociceptive neurons in the spinal cord (113–115) and that altered neuronal responses are accompanied by comparable changes in pain-related behavior. Schneider et al. (115) for example have developed a new animal model on social rejection that mimicks ACE and found that social rejection produces long-lasting effects in pain sensitivity and social behavior that persist into adulthood (115, 116). Moreover, ACE such as parent-child separation have been postulated to be associated with alterations in reproductive traits, prenatal maternal distress, childbirth experience, and labor pain as well as pregnancy outcome (108, 117–119). A recent study showed that childbirth experience in women with childhood sexual abuse was more often frightening and attributed more negatively than in controls (108). Moreover, about half of the women experienced dissociation during childbirth, which was also related to reduced labor pain (108). Additionally, ACE and low socioeconomic status of mothers independently predict low birth weight of the offspring (117, 119).

### Implications for research and clinical practice

ACE are complex etiological marker, that appear to vary on impact in terms of type, timing and severity of maltreatment, together with a wide range of vulnerability and resilience cofactors. In the last years, there has been a welcome increase of research on consequences of ACE on neurobiological, psychological and somatic issues. Overall, the available studies indicate an enduring effect of ACE on mental and physical health throughout the lifespan (67). However, the results need to be treated with some caution, given many differences in study designs (e.g., defining and measuring maltreatment, small sample sizes) or nearly solely cross-sectional studies. Going forward, we need longitudinal studies to better understand how ACE alter brain structure and function and therefore contribute to psychological and somatic consequences. More precisely, future longitudinal studies would allow the valid identification of the influence of important variables including such as age of exposure, type of maltreatment and duration of maltreatment. To sum up, the significant implication of all findings, we summarized in this qualitative review, is that they provide starting points to (1) develop much more explicitly preventive approaches to reduce a child's risk for maltreatment, (2) to implement interventions, that can reduce long-term risk for mental illness in individuals after ACE prior to the emerge of psychopathology and (3) to develop specific treatments for children and adults with psychopathology.

## Author contributions

JH wrote the article, which CS reviewed and approved for publication.

### Conflict of interest statement

The authors declare that the research was conducted in the absence of any commercial or financial relationships that could be construed as a potential conflict of interest.
